# The Effect of Bioactive Glass-Enhanced Orthodontic Bonding Resins on Prevention of Demineralization: A Systematic Review

**DOI:** 10.3390/molecules25112495

**Published:** 2020-05-27

**Authors:** Abdulaziz Alamri, Zainah Salloot, Alaa Alshaia, Maria Salem Ibrahim

**Affiliations:** 1 Preventive Dental Sciences Department, College of Dentistry, Imam Abdulrahman Bin Faisal University, Dammam 31441, Saudi Arabia; absalamri@iau.edu.sa (A.A.); zzsalloot@iau.edu.sa (Z.S.); 2 College of Dentistry, Imam Abdulrahman Bin Faisal University, Dammam 31441, Saudi Arabia; alaalshaia@gmail.com

**Keywords:** bioactive glass, orthodontic bonding resin, demineralization, white spot lesion

## Abstract

At present, bioactive glasses (BAGs) are demonstrating promising results in the remineralization of hard tissues. Their bioactive properties can potentially overcome the demineralization effect accompanying orthodontic treatment. This review aimed to evaluate the effectiveness of bioactive glass enhanced orthodontic bonding resins on enamel remineralization, in addition to their antibacterial, ion release and acid neutralization effect. Four databases (PubMed, MEDLINE, Web of Science and Scopus) were searched. Two hundred and fifty-one full-text articles were screened independently, out of which seven studies satisfied the inclusion criteria. Quality appraisal was performed by two independent reviewers. Methodologies used to assess the anti-demineralization effect included Micro-Computed Tomography, Polarized Light Microscopy and Hardness Testing (Knoop and Berkovich). All seven articles confirmed the superior remineralization effect of BAG orthodontic bonding resins compared to their non-BAG counterparts. A proportional relationship was proved between BAG concentrations and increased anti-demineralization effect. The addition of antibacterial agents to BAG does not necessarily improve its anti-demineralization effect. Although studies have confirmed the effectiveness of BAG orthodontic bonding resins on enamel remineralization, there was a degree of heterogeneity across studies due to the lack of an in vitro studies standardized protocol.

## 1. Introduction

While orthodontic treatment is essential to address functional and esthetic concerns, it serves as an aid to the formation of white spot lesions (WSLs). WSLs are defined as “the first sign of caries lesion on enamel that can be detected with the naked eye” [1,2]. WSLs occur in 45.8% of orthodontic patients [3]. It is more common in maxillary anterior teeth than in the mandibular anterior teeth, which negatively impacts dental esthetics [4]. Fixed orthodontic appliances hinder optimal oral hygiene procedures and create numerous plaque retention sites, which serve as a favorable bacterial growth environment [5]. Subsequently, this reduces the intraoral pH [6]. The drop in pH, in addition to enamel over-etching in some cases, render the tooth surface vulnerable to phosphate (PO_4_) and calcium (Ca) ions loss and thereby advancing WSLs formation [7,8]. 

WSL formation is effectively counteracted by the use of fluoride (F). Fluoride halts the formation of WSLs by interrupting the bacterial metabolic activity, hence the demineralizing bacterial acid production [9]. Fluoride also binds to hydroxyapatite to form fluorapatite, which is more resistant to acidic dissolution than hydroxyapatite. Moreover, fluoride decreases the intracellular pH, thus altering enzymes activity that is essential for bacterial survival [10]. In spite of fluoride’s anti-demineralization effects, its application largely depends on patient cooperation [10]. Incorporating fluoride in Glass Ionomer compounds has been proved effective in preventing WSLs [11], and overcoming the patient cooperation factor. However, the bracket retention force of GIC is lower than that of bracket bonding resins [10]. Prevention of WSLs is also proved effective by the use of casein phosphopeptides-amorphous calcium phosphate (CPP-ACP) containing products [9]. These products work by releasing calcium and phosphate ions in the saliva in instances of pH reduction [9]. Additionally, the disintegration of CPP produces ammonia, which increases the salivary pH, thereby buffering the acidic oral environment [9]. A study proved that depth of carious lesions around orthodontic brackets significantly reduced with the application of CPP-ACP [12]. CPP-ACP is provided in gum, lozenge and cream forms [9]. Hence, like fluoride, its efficacy greatly depends on patient cooperation.

A recent modality for preventing WSLs that does not entail patient cooperation is the incorporation of bioactive glass (BAG) in dental bonding systems [13,14]. BAGs belong to the glass-ceramic biomaterials [13,14]. They are mainly composed of silicon dioxide (SiO_2_), in addition to calcium oxide (CaO), sodium oxide (Na_2_O) and phosphorus pentoxide (P_2_O_5_) [15,16]. The first well-studied formulation of BAG is known as Bioglass^®^ 45S5, which contains 45 wt % SiO_2_, 24.5 wt % Na_2_O, 24.5 wt % CaO and 6 wt % P_2_O_5_ prepared by traditional high-temperature melting, casting and sintering [17,18]. This conventional method of glass synthesis using high thermal treatment affects the properties of BAGs negatively, in terms of bioactivity and porosity [19]. The sol-gel technique was introduced in the early 1970s as an alternative method of glass synthesis [20,21]. It produces highly porous BAGs, which might contribute to the high bioactivity of sol-gel bioactive glasses [22]. BAGs can decrease the likelihood of developing WSLs by different means. It enhances enamel remineralization in five stages [17,23]. First, it contributes to ion exchange, in which BAGs dissolve into an aqueous solution and release sodium ions (Na^+^), calcium ion (Ca^+2^) from the glass network via the exchange in hydrogen ions (H^+^) in the external solution. Second stage is hydrolysis, in which silanol groups (Si-OH) form due to the breakage of silicon-oxygen bond. The following reactions give an overview of the interactions taking place at glass surface–solution interface [24]:

≡Si–OM + H_2_O ⟺ Si–OH + M^+^ + OH^−^

≡Si–Si≡ (glass) + OH^−^ ⟺ ≡Si–OH + ≡Si−O^−^

≡Si–O^−^ + H_2_O ⟺ ≡Si–OH + OH^−^

Next, condensation of sialons occurs, in which the pH rises from the increase in hydroxide ions. The silica network changes its morphology to form a negatively charged gel layer. Afterward, precipitation occurs, in which the formerly developed gel layer functions as a matrix for crystalline precipitation leading to the formation of an amorphous calcium phosphate layer. The final stage is mineralization, in which additional ions are deposited from the surrounding supersaturated solution. Consequently, it promotes the transformation of the calcium phosphate layer into crystalline hydroxyapatite, mimicking the mineral phase of natural tooth structure [17,23,24]. In addition to remineralization, BAGs pose an antibacterial effect primarily driven by the increase in pH, which creates an unfavorable environment for bacterial growth [25,26,27]. The incorporation of variety of antibacterial compounds to BAGs has been proposed in the literature to enhance the combined antibacterial and anti-demineralization properties. These include, but are not limited to, silver, zinc, graphene oxide and gallium [28,29,30].

Albeit proven to reduce enamel demineralization, BAGs possess some fragility. This can be overcome by the enhancement of BAGs with organic-inorganic hybrids during their formulation [31,32]. Organic-inorganic hybrids, provided in polymeric bases, attach to the silica component of BAG through covalent bonds, thereby increasing BAG flexibility and providing better control over the mechanical properties of BAGs [31,32,33].

In addition to BAG fillers, it is noteworthy to mention other bioactive fillers added to orthodontic adhesives to remineralize enamel lesions. Amorphous calcium phosphate (ACP) containing adhesives are bioactive dental materials that release calcium and phosphate ions, which are an important intermediate in hydroxyapatite formation [34]. However, ACP does not form a biomimetic nano-sized apatite layer, as BAG does [35]. Moreover, it showed poor mechanical properties when used to bond orthodontic brackets [36]. Therefore, several glass fillers have been proposed as additives to improve their mechanical performance [37]. 

Researchers have been developing different formulations of BAG to prevent WSLs. A recent systematic review aimed to assess the effectiveness of various forms of bioactive glasses in inducing enamel remineralization, and it concluded that these materials might be more effective in promoting enamel remineralization than fluoride, and CPP-ACP [38]. However, this systematic review includes different modes of application of BAG, which invites a lack in the literature regarding the effectiveness of BAGs in bonding orthodontic appliances. Therefore, the aim of this review was to answer the question whether bioactive glass-enhanced orthodontic bonding resins are superior to non-BAG containing orthodontic bonding resins in preventing demineralization around brackets. The primary objective is to assess the existing evidence that evaluated the ability of different types of bioactive glass-enhanced orthodontic bonding resins in preventing enamel demineralization. The secondary objective is to investigate the evidence that evaluated their antibacterial effect and ions releasing and acid neutralizing abilities.

## 2. Results

### 2.1. Study Selection

**Figure 1:**
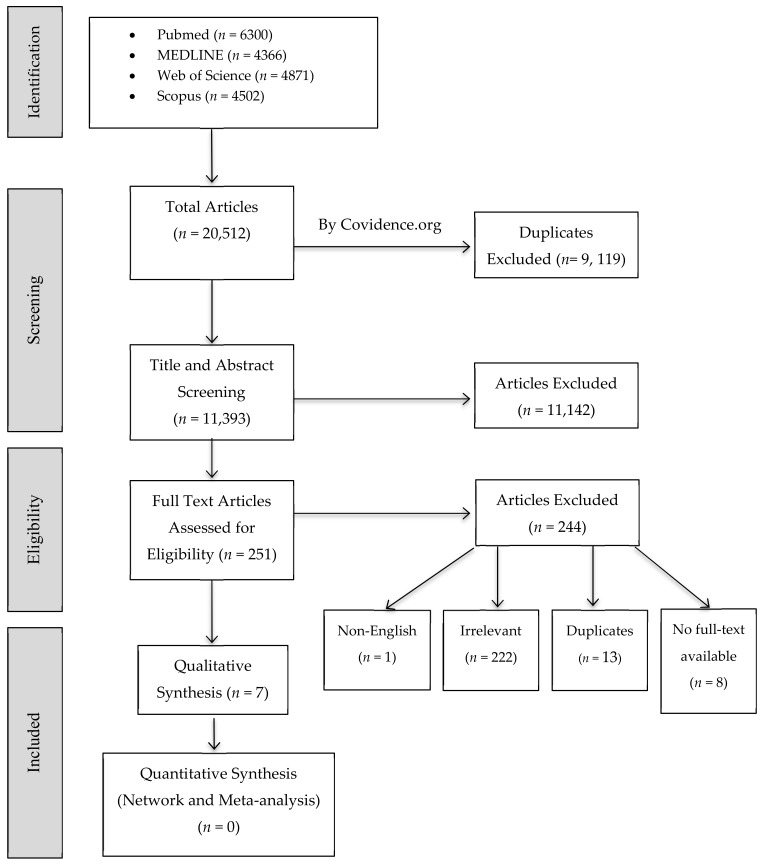
Flow diagram of study screening and selection, PRISMA 2009.

A total number of 20,512 studies identified from the electronic database search as potential relevant were retrieved. After excluding all duplicates, 11,393 studies remained for abstract- and full text-screening. Specific inclusion criteria were applied, and 10,444 were excluded while 251 full-text articles were assessed for eligibility. A total of 7 studies fulfilled the eligibility criteria. The references list for each included article were reviewed and searched for any related article. Out of the 7 included studies, which mainly focused on anti-demineralization effect of BAGs, one study investigated the antibacterial effect of graphene oxide (GO) addition to BAG [29], two studies studied the antibacterial effect and ion release ability of added antibacterial agents (silver oxide (Ag_2_O), zinc oxide (ZnO) [28], gallium oxide (Ga_2_O_3_) [30]) to BAG, and one study investigated ions releasing and acid neutralizing abilities of BAG [39]. A flow diagram of the screening and selection process is demonstrated in Figure 1.

### 2.2. Risk of Bias Appraisal

Out of the seven included studies, one was deemed to have low risk of bias, five were of medium risk and one of high risk (Table 1). Particularly, blinding and sample size calculation parameters were seldom reported across the studies, thereby reducing their quality in this area.

### 2.3. Study Characteristics

#### 2.3.1. BAG and Included Studies Characteristics 

A variation existed in the types and compositions of BAGs used across the included studies. However, all studies assessed BAGs incorporated in orthodontic adhesives; no other mode of application was used. Although a couple of articles used the term ‘bonding agent’ [28,40], the material that was used, had the properties of a bracket adhesive resin. BAGs were added to composite resin adhesives (CharmFilTM Flow; Denkist, Seoul, Korea or TransbondTM XT; 3M, Monrovia, CA, USA) in all studies expect for one study in which BAGs were incorporated in resin modified glass ionomer (RMGIC) [42]. Some studies incorporated additional elements and compounds such as GO [29], Ag_2_O [28], ZnO [28], Ga_2_O_3_ [30] and F [40] to the basic BAG composition of SiO_2_, CaO, P_2_O_5_. Other characteristics of the included studies are reported in Table 2. 

#### 2.3.2. Protocols

Minor variations were noticed between studies in terms of pH-cycling protocols. Generally, all studies prepared teeth samples by cleansing and debridement, protecting tooth surfaces not to be bonded with tape or nail varnish, and etched using different concentrations of phosphoric acid gel. pH-cycling was carried out by immersing samples in demineralizing and remineralizing solutions with certain intervals, after which teeth would be examined for the presence of an anti-demineralization effect by using Micro-computed Tomography scanning (Micro-CT) [28,29,30], Polarized Light Microscopy (PLM) [42], or different hardness tests [39,40]. The anti-demineralization effect of one study [41] was assessed using Fourier Transform Infrared Spectroscopy (FTIR) (Thermo Scientific, Waltham, MA, USA), and Scanning Electron Microscope (SEM) (TESCAN Vega3 LMU, Brno, Kohoutovice, Czech Republic). Details on pH-cycling protocols are mentioned in (Table 3).

To assess the antibacterial effect of BAGs, resin disks of interventional BAG adhesives were cultured with *Streptococcus mutans (S. mutans)* for 24 h [28,30] and 48 h [29]. Following culture, the absorbance was measures at 650 nm using optical density as an assessment method [29,30,31]. To assess ion release, resin disks of interventional BAG adhesives were immersed in distilled water [30,39] or simulated body fluid (Biosesang, Seongnam, Korea) [28] and were assessed after different intervals. Ions release was assessed using coupled plasma optical emission spectrometry [28,30,39]. One study assessed the acid neutralization effect of BAGs [28], in which resin disks of interventional BAG adhesives were immersed in lactic acid solution with a pH of 4.6 at 37 °C, and pH was measured over time by a micro-pH electrode.

#### 2.3.3. Methodologies and Assessment Techniques

Substantial differences in the outcome assessment methodology among the studies were observed. Micro-CT was used to determine the length of enamel remineralization from the end of orthodontic adhesive to the start point of the lesion, where enamel brightness is below 87%. The obtained images were analyzed using an image analysis computer software (image J, National Institutes of Health, Bethesda, MD, USA) [28,29,30]. Hardness testing (HT) to evaluate enamel softening after pH-cycling using a nano-indentation, known as Berkovich indenter (ENT-1100a; Elionix, Tokyo, Japan), was used to measure Berkovich hardness in one study [39].While another study used Knoop indenter (Duramin-5, Struers, Ballerup, Denmark ) to assess the change in enamel microhardness in comparison to the baseline values [40]. One study assessed the anti-demineralization properties by measuring the distance from deepest point of demineralization to the outer surface using PLM (BH2; Olympus, Tokyo, Japan) [42]. Two qualitative assessments techniques using FTIR (Thermo Scientific, Waltham, MA, USA) and SEM (TESCAN Vega3 LMU, Brno, Kohoutovice, Czech Republic) were used by one study. FTIR was used to detect the changes in the tested functional groups before and after demineralization. SEM was used to evaluate enamel microstructure underneath orthodontic brackets after demineralization [41]. Antibacterial properties were assessed using optical density technique in all the studies [28,30,39]. Ion release and acid neutralization were assessed by measuring ion concentration and pH change in solutions, receptively.

#### 2.3.4. Summary of Findings

Summary of the primary outcome findings are presented in Table 4. The addition of different BAG compositions to orthodontic bonding resins showed promising results in preventing enamel demineralization [28,29,30,39,41,42]. It has been proved that anti-demineralizing effect is directly proportional to the filler ratio in a resin matrix [28,29,39]. Lee et al. (2018) found that the anti-demineralization length around the brackets was significantly greater with all three concentrations of BAG resins (BAG@GO 1% (132.4 ± 49 μm), BAG@GO 3% (228.7 ± 135.3 μm) and BAG@GO 5% (218.4 ± 57 μm) than the control group Transbond™ XT Supreme Low-Viscosity (3M, Monrovia, CA, USA) (1.3 ± 0.2), *p* < 0.05 [29]. Similarly, Song et al. (2019), concluded that that the anti-demineralization length was significantly increased with increasing gallium-doped mesoporous bioactive glass nanoparticles (GaMBN) concentration (GaMBN 1% (477.5 ± 260.5 μm), GaMBN 3% (728.4 ± 266.8 μm) and GaMBN 5% (970.3 ± 370.9 μm), *p* < 0.05 [30].

Studies utilizing hardness tests to assess the anti-demineralization effect did not report definite outcome values [39,40]. However, both studies showed superior anti-demineralization effect in comparison to the control materials, as well as a direct proportional relationship between the concentration of BAG in the bonding resins and the anti-demineralization effect.

Summary of antibacterial outcome findings are presented in Table 5. Antibacterial effect of BAG reins was significant in two studies, in which Ag_2_O, ZnO and GO were incorporated in BAG filler [28,29]. Kim et al. (2018) compared the effect of silver and zinc doped BAG against the viability of *Streptococcus mutans* to a BAG without Ag_2_O and ZnO. Non-silver or -zinc doped BAG group showed the highest absorbance. However, it was not significantly different from the control groups; TransbondTM XT (3M, Monrovia, CA, USA) and CharmfilTM Flow (A2 shade, Denkist, Seoul, Korea) [28]. A significant higher antibacterial activity was observed with GO addition to BAG when compared to a commercial control (Transbond™ XT Supreme Low-Viscosity; 3M, Monrovia, CA, USA) [29]. On the other hand, the incorporation of Ga_2_O_3_ to BAG resulted in a non-significant reduction of the bacterial activity [30]. It was notably observed that ion release amounts were proportionally related to the amount of BAG in the orthodontic bonding resins across the studies. Details of ion release values of each study are found in Table 6. In addition, summary of acid neutralization findings as one of the secondary outcomes are presented in Table 7. One study revealed that BAG- 4-methacryloxyethyl trimellitic anhydride/methyl methacrylate-tri-*n*-butyl borane (4-META/MMA-TBB) orthodontic resins are capable of neutralizing acidic environment [39]. Similar to BAG anti-demineralization effect, antibacterial, ion release and pH neutralizing ability was proportional to the concentration of filler content [28,29,39]. 

## 3. Discussion

The purpose of this systematic review was to inquest on how effective BAG orthodontic bonding resins are in battling enamel demineralization around orthodontic brackets. Only seven in vitro studies met the inclusion criteria. This small number of eligible articles, in contrast to the large number initially yielded from search strategies, is partially due to the fact that only studies which tested the anti-demineralization effect on natural teeth were included. The reason behind not accepting articles testing the anti-demineralizing effect on artificial samples, although more abundant, is that natural teeth give a far more realistic picture of what is expected clinically from BAG orthodontic bonding resins. In addition, although a lot of studies investigated the effect of BAG in dental restorative adhesives, the eligibility criteria were set to include only BAG-based orthodontic adhesives. This is because the desired effect of BAG-containing adhesive depends on its total bonded surface area, which in this case is around the dimensions of an orthodontic bracket.

Notwithstanding the foregoing, the included studies in this review carry some variations within. For instance, not all studies confirmed bonding brackets. This heterogeneity in bracket bonding might reflect an exaggerated effect of the BAG-based adhesives tested on non-bonded teeth, as the thickness it was applied in is greater than the thin film applied to brackets realistically. Storage media in which samples were kept prior to testing varied across the articles between distilled water, normal saline and chloramine-T solution. This variation in sample handling is able to significantly influence the results. Kantoor et al. (2015) confirmed that changes in hardness induced by demineralization were significantly associated with different storage media [43]. 

The main protocol, which was utilized to assess the anti-demineralization effect of BAG orthodontic adhesives, was pH-cycling. All included studies employed chemical solutions to carry out pH-cycling, which may not fully mimic the environment of the oral cavity where bacterial acidic challenges may take place. This is in agreement with ten Cate J. (2015), who stated that using a biofilm model which contains bacteria in an environment similar to plaque, paints a more accurate picture of the challenges faced by the oral cavity than chemical pH-cycling [44]. Moreover, in the oral environment, Na^+^ particle in BAGs react with the salivary hydrogen cations (H_3_O^+^) resulting in the release of Ca^+2^ and PO_4_^−^ from their structure, thereby temporarily increasing the salivary pH [38]. This transient pH escalation assists in the Ca^+2^ and PO_4_^−^ precipitation in enamel in order to form the calcium phosphate layer, which later crystalizes to form hydroxyapatite [21,38]. Hence, the similarity of remineralization behavior of BAGs in saliva and in pH-cycling solutions cannot be predicted, given the differences in the compositions and pH between the two.

The effect of an in vitro fourteen-day pH cycle on natural teeth is said to equate to a month’s effect of the oral cavity environment on teeth [41]. While this does provide valuable insight, measuring the anti-demineralization effect of BAGs by pH-cycling can only provide short-term outcomes. Orthodontic treatment lasts for an average of two years [38,45]. Hence, it is difficult to assess the long-term effect of BAGs by relying on the findings lent by a short-term pH-cycling protocol. As such, a method to study the long-term anti-demineralization effect of BAGs is vital to better apply to orthodontic cases.

While appraising included studies for risk of bias, it was observed that in most included studies, examiners were not blinded. This poses a compelling risk of an assessment bias throughout the studies. It was also noted that very few in vitro studies were diligent to perform sample size calculation prior to deciding sample size dimensions. Ideally, sample size must be based on statistical calculation of samples from similar previous studies, 10–15% more than regular sample numbers to compensate for the inconsistencies occurring during testing [46]. Reporting of sample preparation protocol was also marked with inconsistency across the studies. Studies exhibited variegated approaches to prepare samples, which ranged from cleaning with non-fluoridated pumice, curettage to chemical disinfection. 

Judging by the reviewed studies’ outcomes, BAG orthodontic bonding resins showed a higher anti-demineralization effect than non-BAG orthodontic bonding resins. This can be inferred from the reported results of different assessment tools including two types of HT, (ENT-1100a; Elionix, Tokyo, Japan) [39] and (Duramin-5, Struers, Ballerup, Denmark ) [40], Micro-CT [28,29,30], PLM [42] FTIR and SEM [41]. When the anti-demineralization properties of BAGs were measured by Micro-CT, the distance from the point of demineralization to the end of adhesive ranged from 114 to 970 µm [28,29,30]. The highest values were found in GaMBN preparations [30], which might be due to the high percentage of SiO_2_ incorporated in their compositions, since silica is a major determinant of glass bioactivity [15]. This wide range of anti-demineralization effect reflects the intrinsic variations between the identified studies [28,29,30] regarding BAG composition, resin:filler ratio and sample preparation. It is important to highlight the differences in adhesive application procedure. The adhesive was applied to a precisely outlined area in study [28] and [29] with the remaining enamel surface being protected. This is in contrast to Song et al. [30], where the unbonded enamel surface was not protected and thus subjected to inaccuracies in removing excessive adhesive flashes. These flashes might contribute to the higher values of anti-demineralization effect which are inconsistent with other studies [28,29]. Shirazi et al. (2019) was found to be the only study to use RMGI as a base for BAG filler, which resulted in a greater anti-demineralization effect than a non-BAG RMGI [42]. This superior bioactivity is justified by the osmotic gradient generated from RMGI setting reaction that allows the water to be absorbed by the matrix. The increase in water absorption subsequently created an aqueous environment for BAG particles to dissolve and release Na^+^ and Ca^+2^ ions [21,47]. Kohda et al. (2015) found better enamel hardness results in BAG-4-META/MMA-TBB- orthodontic based resin group than a non-BAG containing counterpart [39]. This was concluded from the mean of nanoindentation testing results among the groups without providing a baseline data which is crucial to trace any degree of mineral loss not introduced by the artificial caries challenge leading to a false positive outcome. Therefore, measurement on a cross-sectional level will provide a more detailed and accurate assessment of teeth condition yielding to reliable outcomes. There is an obvious consensus among the studies in dose-response relationship represented by a higher anti-demineralization effect with a higher BAG filler concentration. While assessing the anti-demineralization effect of a BAG orthodontic bonding resin, Firzok et al. (2019) examined the sample under SEM, which provided images of the organization and structure of enamel rods, and by using FTIR, which provided an analysis of the functional groups present in BAGs [41]. Both assessment methods generated qualitative measurements of the remineralization effect of the used BAG orthodontic bonding resin. While its validly demonstrated the anti-demineralization potential of the used adhesives, it deemed it is hard to compare those results with other studies with the same aim, due to the lack of quantitative values.

Several studies added metal ions, such as silver, zinc and gallium, to BAG orthodontic bonding resin in order to combine both antibacterial and demineralization prevention functions [28,29,30]. When 10% Ga_2_O_3_ was added to BAG with 95:5 resin to filler ratio, the enamel lesion started at a much farther distance from the adhesive edge than the addition of 1% Ag_2_O to 90% (wt) resin [28,30]. Both of the antibacterial agents resulted in a superior anti-demineralization effect than the controls. However, the addition of 5% ZnO compromised the anti-demineralization properties of BAG [28]. On the other hand, when GO was added to unidentified composition of BAG to 95% (wt) resin, it resulted in a slightly higher anti-demineralization effect [29]. Therefore, it is very difficult to assign a positive impact of antibacterial agents on the anti-demineralization properties of BAGs. These metal ions exert their antibacterial effect through several mechanisms including changing the bacterial metabolic activities [48,49]. Although lower values of bacterial absorbance were associated with the addition of Ag_2_O, ZnO, Ga_2_O_3_ and GO to BAG [28,29,30], spectrophotometer method of optical density measurement considered the least reliable way for bacterial quantification compared to other methods such as flow cytometry and colony-forming unit counts [50]. 

Three of the included studies performed ion release testing by immersing resin disks composed of their respective intervention and control materials in simulated body fluid (Biosesang, Seongnam, Korea) [28], or distilled water [30,39]. Then, atomic absorption spectrometry method was used. It was difficult to compose a wholesome judgment based on the outcomes of these studies, as outcomes were either not provided in definite quantitative values [28,39], or not supplied with the levels of significance [30]. Nonetheless, Kim et al. (2018) reported that Ca^+2^ and PO_4_^−^ ion release decreased over time. While the reduction in PO_4_^-^ levels is most likely attributed to the deposition of calcium phosphate Ca_3_(PO_4_)_2_, it was peculiar why Ca^+^ levels decreased as well [28]. This is contrary to a study by Brown et al. (2011), who tested the ion release of BAG-Bonds by immersion in simulated body fluid, and found that while PO_4_^−^ levels decreased, Ca^+^ levels increased over time [51]. Acid neutralization effect was tested by Kodha et al. (2015). While no quantitative measurements of changes in the pH were provided, the release of calcium, sodium, silicon, boron ions was reported along with an increase in the pH [39]. This increase is most likely attributed to the release of Na from the tested BAG, which acts as a buffer and is deemed crucial for the precipitation of Ca^+^ and PO_4_^−^ in enamel [38].

Although, the observed trend in the performance of BAG from the presented in vitro studies may correlate to their clinical performance, caution should be taken while inferring the findings. Studies had shown varied degree of correlations between laboratory tests of dental materials and their clinical performance [52]. In vitro models provide simplified settings to study complex phenomena in the oral cavity. The real intraoral environment is not well represented in these laboratory settings [53,54,55]. They may lack some natural protective mechanisms such as the presence of saliva and dental pellicle [56,57,58,59]. In addition, artificial aging systems that simulate the chemical and physical environments of oral cavity can be applied in future in vitro studies to predict the long-term performance of these materials [54].

Overall, there seemed to be a lack of standardized protocols to be followed while conducting in vitro study tests, which exposes studies to greater risk of bias. The development of a standardized protocol to follow while attempting an in vitro study is essentially needed, as it can readily tackle the recurring inconsistencies, minimize risk of bias and provide more homogenous study characteristics of future in vitro studies.

## 4. Materials and Methods

### 4.1. Research Question

This review followed the PRISMA guidelines for systematic reviews and meta-analysis [60]. Review protocol of articles was pre-determined by the reviewers; however, it is an unpublished protocol. The review question was: Are bioactive glass (BAG) enhanced orthodontic bonding resins superior to non-BAG enhanced orthodontic bonding resins in preventing demineralization around orthodontic brackets? 

### 4.2. Definitions 

BAGs are silicon dioxide compounds, primarily consisting of SiO_2_, CaO and P_2_O_5_ [15,16]. Orthodontic bonding resins are resin materials involved in orthodontic bracket bonding, which include primers, bonding agents and bonding resins. Demineralization is a process in which minerals are lost from hydroxyapatite crystals of enamel [61].

### 4.3. Search Strategy

Detailed search strategies of four electronic databases were developed and searched by two authors. PubMed was queried for published articles on April 3, 2020 irrespective of language and date and resulted in 6773 references. MEDLINE, Web of Science and Scopus databases were searched on April 3–4, 2020 and 4366, 4871 and 4502 potentially relevant studies were found, respectively. PubMed search was explained in detailed in Table 8 as an example for the search strategy. At this stage, there was no date or language restraints. Literature search citations were imported to Covidence online platform to remove duplications and carry the screening process. 

### 4.4. Inclusion and Exclusion Criteria

Following our selection criteria, articles included in this review were in vitro studies. Samples in the included studies consisted of natural teeth. Intervention materials in included studies consisted of BAG containing resins used for orthodontic bracket bonding purposes. Studies testing BAG resins with non-orthodontic uses were excluded. Similarly, studies that did not measure the anti-demineralization effect of BAG orthodontic bonding resins were excluded. Commercial bonding resins which do not contain BAG in their compositions were excluded. Compositions on commercial bonding resins were often retrieved from their corresponding safety data sheets (SDSs). If a material’s SDS was not available, then the composition would be researched in the literature.

### 4.5. Studies Screening and Selection

Two independent reviewers, who were not blinded to the identity of authors, journal or the results of the studies, carried out the studies selection procedure. It consisted of title and abstract screening then full text screening. Studies were deemed not eligible if one of the exclusion criteria was met. The full text of studies considered by either reviewer eligible for inclusion were retrieved and screened. Disagreements among reviewers were solved by joint discussion between two reviewers.

### 4.6. Data Extraction

Two independent authors recorded desired information from included studies using a customized data collection form, constructed on Microsoft Excel (2020). The data extraction form consisted of open and close-ended questions to assess qualitative and quantitative information. The following information were extracted: Sample characteristics including number, type of teeth and preparation. Intervention and control characteristics, with a focus on composition, and BAG content, type and ratios. Demineralization-remineralization protocol, which includes composition and pH of solutions, immersion time and intervals. Information regarding the secondary outcomes included type of cultured bacteria, culturing condition for measuring the antibacterial effect. Ultimately, data concerning the outcome assessment methods, outcome values and units of measurement were extracted. 

### 4.7. Quality Assessment

Included studies were appraised for risk of bias by three independent reviewers, using a well-accepted quality assessment tool adapted from several studies [38,62,63]. Sampling bias was appraised by assessing whether a study examined teeth for being sound and caries free. Sampling bias was also measured by assessing whether or not teeth underwent sample size calculation, preparation and randomization. Sample size calculation was reported when a formula justifying the number of samples was provided. Sample preparation was reported when a study described how teeth were handled, cleaned and prepared for pH cycling. Sample randomization entails that teeth were randomly assigned to intervention and control groups. Studies were also appraised on having control groups, and blinding of examiners, in which it describes whether or not an examiner is aware of the applied intervention when measuring the outcome. The tool also assessed the presence of definitive values and statistical analysis. Reporting bias was described when an article did not provide definitive values after measuring outcomes, and only supplied outcome values in graphs or in text. However, this parameter was not applicable when a study only utilized qualitative measurement methods. Lack of analysis was described when a study did not contain any quantitative values to undergo analysis. If a parameter was clearly stated in an article, the article would have a “Yes” on said parameter. If a parameter was not mentioned in an article, the article would have a “No” on said parameter. If a parameter was reported while measuring a secondary outcome but not while measuring the primary outcome, the article would also have a No on said parameter. Articles containing one to three parameters were considered to have a high risk of bias. Articles containing four to five and six to eight parameters were considered to have a medium and low risk of bias, respectively.

### 4.8. Assessment of Heterogeneity

Reviewers developed data extraction forms to record variables related to the interventional, methodological and statistical heterogeneity of the included studies. Interventional heterogeneity was assessed by qualitatively examining the differences in studies’ interventions composition formulations such as resin matrix, fillers percentage and incorporation of other agents such as antibacterial agents. Methodological heterogeneity was examined by comparing the different studies’ setting, samples’ preparation protocols, demineralization protocols, primary outcome assessment, studies’ overall all risk of bias. 

### 4.9. Data Synthesis 

We planned to perform a quantitative meta-analysis utilizing a fixed-effect model if we found an I_2_ statistics at or below 50% with no significant clinical and methodological heterogenicities. On the other hand, if an I_2_ statistics is found to be above 50% with no significant clinical and methodological heterogenicities, a random-effect model was planned to be applied. However, if we found a significant statistical heterogeneity and observed clinical and methodological heterogenicities, a meta-analysis will not be conducted. In all situations described above, a qualitative synthesis will include descriptions of the outcomes, similarities and differences in the methodologies employed, interventions characteristics and any additional relevant information.

## 5. Conclusions

In conclusion, BAG orthodontic bonding resins showed significantly superior anti-demineralization effect than non-BAG orthodontic bonding resins. The addition of antibacterial agents to BAG does not necessarily improve its anti-demineralization effect. This conclusion is based on laboratory and in vitro studies. It should be carefully interpreted as the clinical performance of these materials needs further investigations and clinical trials. Development of a standardized protocol to follow while attempting an in vitro study is essential to minimize risk of bias and provide more homogenous studies. In turn, this would increase the feasibility of producing more uniform systematic reviews and meta-analyses.

## Figures and Tables

**Table 1:** Risk of bias appraisal.

	Sampling Bias				Assessment Bias		Reporting Bias		Risk of Bias
Study	Caries-Free Teeth	Sample Size Calculation	Sample Preparation	Sample Randomization	Presence of Control Group	Blinding	Definitive Values	Quantitative Analysis	
Manfreda et al., 2013 [40]									Medium
Kohda et al., 2015 [39]									Medium
Kim et al., 2018 [28]									High
Lee et al., 2018 [29]									Medium
Firzoka et al., 2019 [41]							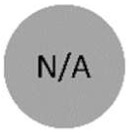		Medium
Shirazi et al., 2019 [42]									Low
Song et al., 2019 [30]									Medium


Yes 

No 
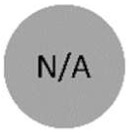
 Not Applicable Parameter.

**Table 2:** Characteristics of included studies.

Study	Group Sample Size	Teeth Type	Teeth Storage Media	Sample Preparation	BAG Composition	BAG Synthesis Method	BAG Particle Size	BAG Ratio/ Weight	Control Groups	Tested Groups
Manfreda et al., 2013 [40]	10	Human, non-carious third molars	0.5% Chloramine-T solution at 4 °C	Cleansing with non-fluoridated pumice and water using a prophylaxis cup	62BAG 65BAG 81BAG 85BAG	Sol-gel	62BAG: 75 m^2^/g 65BAG:144 m^2^/g 81BAG:320 m^2^/g 85BAG:268 m^2^/g	62BAG: 58:100 65BAG: 49:100 81BAG: 37:100 85BAG: 33:100 (BAG: Monomer)	TXT	62BAG-Bond 65BAG-Bond 81BAG-Bond 85BAG-Bond
Kohda et al., 2015 [39]	10	Human, non-carious, upper premolars		Cleansing with non-fluoridated pumice and water using a prophylaxis cup	45.0% SiO_2_ + 24.5% Na_2_O + 24.5% CaO + 6.0% P_2_O_5_	Melting and grinding	100 µm	0, 10, 20, 30, 40 or 50%	PMMA powder containing 0% BAG + 4-META/MMA + TBB	PMMA powder containing 10, 20, 40 or 50% BAG + 4-META/MMA + TBB
Kim et al., 2018 [28]	Not Clear	Human premolar			Silver- and Zinc-doped BAGs: A0 A1 A1Z5 Z5	Sol-gel		10 or 15%	CF TXT	CF + 10% A0 CF + 10% A1 CF + 10% A1Z5 CF + 15% A1Z5 CF + 10% Z5
Lee et al., 2018 [29]	9	Human, non-carious, upper premolars		Cleansing with non-fluoridated pumice and water using a prophylaxis cup	BAG@GO	Sol-gel		1, 3 or 5%	LV	LV + 1, 3 or 5% BAG@GO
Firzoka et al., 2019 [41]	30	Human, non-carious, premolars	Normal Saline at 4 °C	Disinfecting with 0.5% Chloramine-T solution for 24 h	F-BGC-1 BGC-1 F-BGC-2 BGC-2	Sol-gel	F-BGC-1: 69.89 m^2^/g BGC-1: 71.08 m^2^/g F-BGC-2: 65.45 m^2^/g BGC-2: 65.34 m^2^/g	5%	TXT	TXT + F-BGC-1 TXT + BGC-1 TXT + F-BGC-2 TXT + BGC-2
Shirazi et al., 2019 [42]	20	Human, non-carious, premolars	0.1% Thymol solution for one week then stored in distilled water at 6 °C	- Curettage - Cleansing with pumice and water using a prophylaxis cup				30%	TXT Fuji II LC	Fuji II LC + 30% BAG
Song et al., 2019 [30]	10	Human, non-carious, premolars		- Cleansing with pumice. - Etching of bonding area with 35% phosphoric acid gel for 15 s	Gallium-Doped BAG; GaMBN	Modified sol-gel	404.09 m^2^/g	1, 3 or 5%	CF	CF + 1, 3, or 5% GaMBN

62BAG: 62 mol % SiO_2_ + 31 mol % CaO + 4 mol % P_2_O_5_ + 1 mol % B_2_O_3_ + 3 mol % F; 65BAG: 65 mol % SiO_2_ + 31 mol % CaO + 4 mol % P_2_O_5_ + 0 mol % B_2_O_3_ + 0 mol % F; 81BAG: 81 mol % SiO_2_ + 11 mol % CaO + 4 mol % P_2_O_5_ + 1 mol % B_2_O_3_ + 3 mol % F; 85BAG: 85 mol % SiO_2_ + 11 mol % CaO + 4 mol % P_2_O_5_ + 0 mol % B_2_O_3_ + 0 mol % F; CF: CharmfilTM Flow (Denkist, Seoul, Korea); A0: 58% SiO_2_ + 33% CaO + 9% P_2_O_5_; A1: 58% SiO_2_ + 32% CaO + 9% P_2_O_5_ + 1% Ag_2_O; A1Z5: 58% SiO_2_ + 27% CaO + 9% P_2_O_5_ + 1% Ag_2_O + 5% ZnO; Z5: 58% SiO_2_ + 28% CaO + 9% P_2_O_5_ + 5% ZnO; TXT: TransbondTM XT (3M, Monrovia, CA, USA); 62BAG-Bond: Ethoxylated bisphenol A dimethacrylate + BisGMA + 62BAG + 0.4 wt % camphoroquinone + 0.8 wt % ethyl 4-dimethylaminobenzoate; 65BAG-Bond: Ethoxylated bisphenol A dimethacrylate + BisGMA + 65BAG + 0.4 wt% camphoroquinone + 0.8 wt % ethyl 4-dimethylaminobenzoate; 81BAG-Bond: Ethoxylated bisphenol A dimethacrylate + BisGMA + 81BAG + 0.4 wt % camphoroquinone + 0.8 wt % ethyl 4-dimethylaminobenzoate; 85BAG-Bond: Ethoxylated bisphenol A dimethacrylate + BisGMA + 85BAG + 0.4 wt % camphoroquinone + 0.8 wt % ethyl 4-dimethylaminobenzoate; PMMA: Polymethyl methacrylate; 4-META/MMA-TBB: 4-methacryloxyethyl trimellitic anhydride/methyl methacrylate-tri-*n*-butyl borane; LV: Transbond™ XT Supreme Low-Viscosity (3M, Monrovia, CA, USA); BAG@GO: Graphene Oxide containing BAG; F-BGC-1: 46 mol % SiO_2_ + 23.5 mol % CaO (Source: Ca(OH)_2_) + 23 mol % Na_2_O + 2.5 mol % P_2_O_5_ + 5 mol % NaF; BGC-1: 46 mol % SiO_2_ + 28.5 mol % CaO (Source: Ca(OH)_2_) + 23 mol % Na_2_O + 2.5 mol % P_2_O_5_ + 0 mol % NaF; F-BGC-2: 46 mol % SiO_2_ + 23.5 mol % CaO (Source: Ca(NO_3_)_2_.4H_2_O) + 23 mol % Na_2_O + 2.5 mol % P_2_O_5_ + 5 mol % NaF; BGC-2: 46 mol % SiO_2_ + 28.5 mol % CaO (Source: Ca(NO_3_)_2_.4H_2_O) + 23 mol % Na_2_O + 2.5 mol % P_2_O_5_ + 0 mol% NaF; Fuji II LC: Resin modified glass ionomer (GC Corp., Tokyo, Japan); GaMBN: 70 mol % SiO_2_ + 15 mol % CaO + 5 mol % P_2_O_5_ + 10 mol % Ga_2_O_3._

**Table 3:** Remineralization assessment methodology.

Study	Sample Preparation	pH Cycling Protocol				Outcome Measurement Method
		Demineralization	Remineralization	Days of Cycle Repetition	Notes	
Manfreda et al., 2013 [40]	- Application of acid-resistant varnish leaving a 1-mm rim of exposed enamel surrounding the bracket - Etching of bonding area with 37% phosphoric acid gel for 30 s	For 6 h, teeth were immersed in 40 mL demineralization solution consisting of 2.0 mM Ca, 2.0 mM PO_4_ and 0.075 mM CH_3_COOH at pH 4.4.	For 18 h, teeth were immersed in 40 mL of remineralization solution consisting of 1.5 mM Ca, 0.9 mM PO_4_, 0.1 5 M KCl and 20 mM C_2_H_6_AsNaO_2_ at pH 7.	14	- Fresh solutions were used each week. - -Teeth were rinsed with deionized water between the solutions. - The cycle was repeated 5 days a week, with teeth remaining in artificial saliva during week-ends.	Knoop Microhardness Testing: Hardness was measured using Knoop indenter.
Kohda et al., 2015 [39]	- Application of acid-resistant nail varnish leaving a 1-mm rim of exposed enamel surrounding the bracket - Etching of bonding area with 20% phosphoric acid gel for 20 s	For 4 h, teeth were immersed in 2 mL demineralization solution consisting of 2 mM CaCl_2_ and 2 mM NaH_2_PO_4_ with 50 mM CH_3_COOH) at pH 4.55.	For 20 h, teeth were immersed in 2 mL remineralization solution consisting of 2 mM CaCl_2_ and 2 mM NaH_2_PO_4_ with 0.1 M of NaOH at pH 6.8.	14		Nano-indentation Testing: Hardness was measured using Berkovich indenter.
Kim et al., 2018 [28]	- Nail varnish coating of non-bonding tooth surfaces - Etching of bonding area with 35% phosphoric acid gel for 30 s	For 6 h, teeth were immersed in a demineralization solution consisting of 2.0 mM Ca(NO_3_)_2_·4H_2_O, 2.0 mM KH_2_PO_4_ and 75.0 mM CH_3_COOH at pH of 4.4.	For 18 h, teeth were immersed in a remineralization solution consisting of 20.2 mM C_2_H_12_AsNaO_5,_ 1.5 mM, Ca(NO_3_)_2_·4H_2_O, 0.9 mM KH_2_PO_4_ and 130 mM CaCl_2_ at pH 6.8.	14	- Fresh solutions were used each week. - Teeth were rinsed with deionized water between the solutions.	Micro-CT Scanning: Intensity histograms were used to measure the lesion depth, remineralization zone width and mineral loss.
Lee et al., 2018 [29]	- Tape covering of non-bonding surfaces - Etching of bonding area with 35% phosphoric acid gel for 30 s	For 6 h, teeth were immersed in a demineralization solution (Biosesang, Seoul, Korea)	For 18 h, teeth were immersed in an anti-demineralization solution (Biosesang, Seoul, Korea)	14	- Fresh solutions were used each week. - Teeth were rinsed with deionized water between the solutions.	Micro-CT Scanning: Brightness histograms were used to measure the anti-demineralization length.
Firzoka et al., 2019 [41]	- Etching of bonding area with 37% phosphoric acid gel for 20 s	For 6 h, teeth were immersed in 4 mL demineralization solution consisting of CaCl_2,_ Na_3_PO_4_, CH_3_COOH, KOH and thymol crystals at pH 4.4.	For 18 h, teeth were immersed in 4 mL remineralization solution consisting of CaCl_2,_ Na_3_PO_4_, KCl and thymol crystals at pH 7.	14		- Fourier Transform Infrared Spectroscopy (FTIR): Spectra range of 4000–600 cm^−1^ was used to identify changes in the functional groups. - Scanning Electron Microscopy (SEM): Qualitative analysis was used.
Shirazi et al., 2019 [42]	- Nail varnish coating of non-bonding surfaces - Etching of bonding area with 37% phosphoric acid gel for 30 s	For 6 h, teeth were immersed in 10 mL demineralization solution consisting of 2.2 mM CaCl_2_, 50 M CH_3_COOH and 2.2 mM KH_2_PO_4_, 35.78 mL of 1 M C_6_H_8_O_7_, 14.22 mL of 1 M C_2_H_3_NaO_2_, 0.0022 M KH_2_PO_4_ and 0.0022 M CaCl_2_ at pH 4.3.	For 18 h, teeth were immersed in 10 mL remineralization solution consisting of 1.5 mM CaCl_2_, 150 mM KCl and 0.9 mM KH_2_PO_4_ at pH 7.	21	Fresh solutions were used each week.	Polarized Light Microscopy: Depth of the lesion was measured.
Song et al., 2019 [30]	- Cleansing with pumice - Etching of bonding area with 35% phosphoric acid gel for 15 s	For 6 h, teeth were immersed in 500 mL demineralization solution consisting of 2 mM Ca(NO_3_).4H_2_O, 2 mM KH_2_PO_4_ and 75 mM CH_3_COOH at pH 4.4.	For 18 h, teeth were immersed in 500 mL remineralization solution consisting of CH_3_COOH.4H_2_O, 0.9 mM KH_2_PO_4,_ 130 mM KCl and 20.2 mM NaC_2_H_6_AsO_2._3H_2_O at pH 7.	14	- Fresh solutions were used each week. - Teeth were rinsed with deionized water between the solutions.	Micro-CT Scanning: Brightness histograms were used to measure the anti-demineralization length.

**Table 4:** Primary outcome findings.

Assessment Tool	Study	Intervention (Mean ± SD)	Control (Mean ± SD)	Summary of Results
Micro-computed Tomography	Kim et al., 2018 [28]	Lesion Depth: CF + 10% A0: (0.17 ± 0.018 µm) CF + 10% A1: (0.095 ± 0.014 µm) CF + 10% A1Z5: (0.091 ± 0.017 µm) CF + 10% Z5 (0.073 ± 0.011 µm) Mineral Loss: CF + 10% A0: (198.95 ± 33.42) CF + 10% A1: (219.04 ± 63.73) CF + 10% A1Z5: (183.15 ± 48.2) CF + 10% Z5 (113.95 ± 21.09) Remineralization Zone Width: CF + 10% A0: (0.292 ± 0.088 µm) CF + 10% A1: (0.257 ± 0.058 µm) CF + 10% A1Z5: (0.236 ± 0.56 µm) CF + 10% Z5 (0.345 ± 0.024 µm)	Lesion Depth: CF: (0.093 ± 0.025 µm) * TXT: (0.099 ± 0.022 µm) * Mineral Loss: CF: (219.08 ± 64) * TXT: (172.83 ± 43.79) * Remineralization Zone Width: CF: (0.143 ± 0.02 µm) * TXT: (0.048 ± 0.026 µm) *	- Ag- or Zn-doped BAG containing bonding agents promoted more enamel remineralization when compared to non-BAG containing orthodontic bonding agents. - CF+Z5-10 showed the least mineral loss and lesion depth among the groups.
	Lee et al., 2018 [29]	LV + 1% BAG@GO: (132.4 ± 49 µm) LV + 3% BAG@GO: (228.7 ± 135.3 µm) LV + 5% BAG@GO: (218.4 ± 57 µm)	LV (1.3 ± 0.2 µm) *	- BAG-GO containing adhesives showed better results than the commercial control adhesive based on anti-demineralization results. - A direct proportional relationship between GO concentration and anti-demineralization effect was observed.
	Song et al., 2019 [30]	CF + 1% GaMBN: (477.5 ± 260.5 µm) CF + 3% GaMBN: (728.4 ± 266.8 µm) CF + 5% GaMBN: (970.3 ± 370.9 µm)	CF (53.7 ± 22.2 µm) **	- GaMBN containing orthodontic resins were effective in preventing enamel demineralization in comparison to the commercial control. - A direct proportional relationship between GaMBN concentration and anti-demineralization effect was observed.
Hardness Testing	Manfreda et al., 2013 [40] ᵻ	−	−	All the BAG containing orthodontic bonding agents (BAG-Bonds) outperformed the commercial control in regard to enamel hardness surrounding the brackets.
	Kohda et al., 2015 [39]	Values were provided in a supplemental document.	Values were provided in a supplemental document.	All the BAG containing 4META/MMA-TBB-based resins outperformed the commercial control in regard to enamel hardness surrounding the brackets.
Polarized Light Microscopy	Shirazi et al., 2019 [42]	Fuji II LC + 30% BAG (73.8 ± 22.29 µm)	TXT (182.98 ± 20.69 µm) * Fuji II LC (118.08 ± 29.42 µm) *	BAG containing RMGIC showed higher ability to prevent demineralization by a significant reduction in demineralization depth under orthodontic brackets in comparison to the commercial controls.
Fourier Transform Infrared and Spectroscopy Scanning Electron	Firzok et al., 2019 [41]ᵻ	−	−	- Ag- or Zn-doped BAG containing bonding agents promoted more enamel remineralization when compared to non-BAG containing orthodontic bonding agents. - CF+Z5-10 showed the least mineral loss and lesion depth among the groups.

* Significant difference between experimental and control groups (*p* < 0.05). ** Significant difference between experimental and control groups (*p* < 0.001). ᵻ No definitive values were reported.

**Table 5:** Secondary outcome findings—antibacterial effect.

Antibacterial Effect				
Assessment Tool	Study	Intervention (Mean ± SD)	Control (Mean ± SD)	Summary of Results
Optical Density	Kim et al., 2018 [28]	CF + 10% A0: (0.22 OD at 620 nm) CF + 10% A1: (0.3 OD at 620 nm) CF + 10% A1Z5: (0.22 at OD 620 nm) CF + 15% A1Z5: (0.29 at OD 620 nm) CF + 10% Z5 (0.28 at OD 620 nm)	CF: (0.38 OD at 620 nm) * TXT: (0.35 OD at 620 nm) *	All interventional resins showed significantly lower absorbance values than control resins.
	Lee et al., 2018 [29]	In 24 h,	In 24 h,	- After 24 h, the antibacterial effect of the control group was significantly lower than the interventional groups. - After 48 h, the difference in the antibacterial effect was not significantly different between the interventional and control groups. however, a proportional relationship was demonstrated between the antibacterial effect of the interventional groups and different concentrations of BAG@GO.
		LV + 1% BAG@GO: (2.1 ± 0.2% at 620 nm) LV + 3% BAG@GO: (3 ± 2.6% at 620 nm) LV + 5% BAG@GO: (4.2 ± 2.8% at 620 nm)	LV (67.2 ± 14.5% at 620 nm) *	
		In 48 h,	In 48 h,	
		LV + 1% BAG@GO: (0.6 ± 0.2% at 620 nm) LV + 3% BAG@GO: (0.6 ± 0.1% at 620 nm) LV + 5% BAG@GO: (0.5 ± 0.1% at 620 nm)	LV (62 ± 9.8% at 620 nm) *	
	Song et al., 2019 [30] ᵻ	−	−	- There was no significant difference in *S. mutans* viability between the interventional and control groups. - However, an inversely proportional relationship was demonstrated between the viability of *S. mutans* and the concentration of GaMBN in the resin.

* Significant difference between experimental and control groups (*p* < 0.05) ᵻ No definitive values were reported.

**Table 6:** Secondary outcome findings—ions release.

Ion Release						
Assessment Tool	Study	Intervention (Mean ± SD)			Control (Mean ± SD)	Summary of Results
Ion Release	Kim et al., 2018 [28]ᵻ	−			−	- In all groups, Ca and PO_4_ ions concentrations decreased after 72 h. - In 10% A1 and 10% A1Z5 groups, Ag was detected after 6 h of immersion in simulated body fluid and continued to increase in concentration until 840 hours of immersion. - After 840 h of immersion, minute traces of Zn were detected.
	Kohda et al., 2015 [39]ᵻ	−			−	- In 3 months of immersion, Ca, Na, Si and B were regularly released. - A proportional relationship was found between the amount of ions release and the BAG content in the resins.
	Song et al., 2019 [30]	In 1 day	In 7 days	In 14 days	Barely released	- Ions were barely released by the commercial control. - A proportional relationship was found between the amount of ions release and the GaMBN content in the resins.
		CF + 1% GaMBN	CF + 1% GaMBN	CF + 1% GaMBN		
		Ca: (3.8 ± 0.1) ppm *	Ca: (7.2 ± 0.2) ppm *	Ca: (7.1 ± 0.1) ppm *		
		P: (0.4 ± 0) ppm *	P: (1.2 ± 0) ppm *	P: (0.8 ± 0) ppm *		
		Ga: (0.2 ± 0) ppm *	Ga: (1.5 ± 0) ppm *	Ga: (2.1 ± 0.1) ppm *		
		CF + 3% GaMBN	CF + 3% GaMBN	CF + 3% GaMBN		
		Ca: (6.4 ± 0.1) ppm *	Ca: (17.8 ± 0.8) ppm *	Ca: (16.6 ± 0.1) ppm *		
		P: (1 ± 0) ppm *	P: (3.3 ± 0.1) ppm *	P: (3.4 ± 0.1) ppm *		
		Ga: (0.6 ± 0) ppm *	Ga: (4.8 ± 0.3) ppm *	Ga: (5.7 ± 0.5) ppm *		
		CF + 5% GaMBN	CF + 5% GaMBN	CF + 5% GaMBN		
		Ca: (6.3 ± 0.2) ppm *	Ca: (19.5 ± 0.3) ppm *	Ca: (27.2 ± 0.8) ppm *		
		P: (1.3 ± 0.1) ppm *	P: (4.5 ± 0.1) ppm *	P: (6.4 ± 0.1) ppm *		
		Ga: (0.5 ± 0) ppm *	Ga: (6.7 ± 0.3) ppm *	Ga: (6.7 ± 0.1) ppm *		

*p-value was not reported. ᵻ No definitive values were reported.

**Table 7:** Secondary outcome findings—acid neutralization.

Acid Neutralization				
Assessment Tool	Study	Intervention (Mean ± SD)	Control (Mean ± SD)	Summary of Results
pH Change	Kohda et al., 2015 [39] ᵻ	−	−	- An increase in the pH was demonstrated with BAG containing resins. - There was a proportional relationship between pH increase and BAG content in the resins.

ᵻ No definitive values were reported.

**Table 8:** Search strategy in PubMed.

Search	Terms
1	(“Resins, Synthetic”[Mesh] OR “Dental Bonding”[Mesh]) OR “Dental Materials”[Mesh])
2	(“bioactive glass”[tw] OR “bioglass”[tw] OR “bioceramic”[tw])
3	(“Tooth Demineralization”[Mesh] OR “Tooth Remineralization”[Mesh])
4	(“Remineralization”[tw] OR “Remineralisation”[tw], OR “Demineralization”[tw], OR “Demineralisation”[tw])
5	#1 OR #2
6	#3 OR #4
7	#5 AND #6
